# Dr. Gregory Doyle

**Published:** 1913-09

**Authors:** 


### OBITUARIES.

***Readers are requested to report promptly the death of any physician in Western New York, or former residents of this region, or graduates of any medical school in Western New York and to call the attention of the families of the deceased, our desire to publish adequate obituary notices.***

---

Dr. Gregory Doyle, N. Y. University, 1865, died at his home in Syracuse, July 23. He was born in Ireland, March 28, 1840, emigrated the next year, graduated in arts at Niagara University in 1860 and received the honorary degree of LL. D. from the same institution in 1898. After graduating in medicine he settled in Syracuse. He was a member of the U. S. Pension Board, 1885-89, Surgeon and Major in the National Guard, 1872-90, Health Commissioner of Syracuse, 1899-1904. He was a prominent surgeon and author of Incidents of European Travel, 1910, as well as a contributor to current medical literature.

